# Genomic alterations associated with pseudoprogression and hyperprogressive disease during anti-PD1 treatment for advanced non-small-cell lung cancer

**DOI:** 10.3389/fonc.2023.1231094

**Published:** 2023-11-09

**Authors:** Rui Zhou, Fan Tong, Yongchang Zhang, Ruigang Zhang, Yawen Bin, Sheng Zhang, Nong Yang, Xiaorong Dong

**Affiliations:** ^1^ Cancer Center, Union Hospital, Tongji Medical College, Huazhong University of Science and Technology, Wuhan, China; ^2^ Institute of Radiation Oncology, Union Hospital, Tongji Medical College, Huazhong University of Science and Technology, Wuhan, China; ^3^ Department of Medical Oncology, Lung Cancer and Gastrointestinal Unit, Hunan Cancer Hospital/The Affiliated Cancer Hospital of Xiangya School of Medicine, Central South University, Changsha, China

**Keywords:** next-generation sequencing analysis, pseudoprogression, hyperprogressive disease, anti-PD1 treatment, non-small cell lung cancer

## Abstract

**Introduction:**

This study aimed to elucidate the relationship between dynamic genomic mutation alteration and pseudoprogression (PsPD)/hyperprogressive disease (HPD) in immunotherapy-treated advanced non-small-cell lung cancer (NSCLC), to provide clinical evidence for identifying and distinguishing between PsPD and HPD.

**Method:**

Patients with advanced NSCLC who were treated with anti-PD1 were enrolled. Whole blood was collected at baseline and post image progression. Serum was separated and sequenced using 425-panel next-generation sequencing analysis (NGS).

**Results:**

NGS revealed that not only single gene mutations were associated with PsPD/HPD before treatment, dynamic monitoring of the whole-blood genome mutation spectrum also varied greatly. Mutational burden, allele frequency%, and relative circulating tumor DNA abundance indicated that the fold change after image progression was much higher in the HPD group.

**Discussion:**

The gene mutation profiles of PsPD and HPD not only differed before treatment, but higher genome mutation spectrum post image progression indicated true disease progression in patients with HPD. This suggests that dynamic whole-genome mutation profile monitoring as NGS can distinguish PsPD from HPD more effectively than single gene detection, providing a novel method for guiding clinical immune treatment.

## 1 Introduction

The widespread application of immune-checkpoint inhibitors (ICIs) in the treatment of non-small-cell lung cancer (NSCLC) has substantially prolonged patients’ overall survival (1). However, they are not always effective for the entire population. Some studies have reported that programmed death-ligand 1 (PDL1), tumor mutational burden (TMB), mismatch repair, and CD8^+^T cells may be potential biomarkers for efficacy prediction, whereas Janus kinase 1 (JAK1), JAK2, and beta-2-microglobulin truncation may be predictors of primary resistance (2–4). In addition to the uncertain prediction of efficacy, the immunotherapy response also differs from that of classical radiotherapy and chemotherapy. Two distinct and atypical patterns of response to ICIs are pseudoprogression (PsPD) and hyperprogressive disease (HPD). PsPD is characterized by an increase in tumor size or the appearance of new lesions after ICI treatment, followed by tumor regression (5). HPD represents a novel pattern of progression, with an unexpected and rapid increase in both tumor volume and rate (6). The conventional imaging-based efficacy evaluation model may lead to tremendous bias, engendering confusion for clinicians. Hence, iRECIST has been redefined to differentiate response patterns between immunotherapy, chemotherapy, and radiotherapy (7). However, precise methods for distinguishing between PsPD and HPD are still lacking in clinical practice, necessitating the formulation of appropriate prediction strategies in such cases.

Next-generation sequencing (NGS), also termed high-throughput or massively parallel sequencing, is a technology that facilitates simultaneous and independent sequencing of thousands to billions of DNA fragments. This method not only detects the mutations in a single gene but also analyzes the total abundance and characteristics of all gene mutations (8). Continuous NGS of patient samples before and after treatment can provide information regarding the changes in gene profiles resulting from immunotherapy. Tissue detection is accurate, but continuous testing is encumbered by the invasiveness of the specimen acquisition procedure. Blood NGS testing can partially replace tissue sample testing, as it enables continuous testing owing to the ease of obtaining specimens (9, 10).

To date, no study has examined the relationship between dynamic genomic alterations in blood and the occurrence of PsPD and HPD in patients with NSCLC who undergo immunotherapy. To date, no study has examined the relationship between dynamic genomic alterations in blood and the occurrence of PsPD and HPD in patients with NSCLC who undergo immunotherapy. It provides a transformative and practical method for predicting the clinical response of NSCLC to immunotherapy and offers individualized scheme selection of immunotherapy based on dynamic NGS detection.

## 2 Materials and methods

### 2.1 Patients

This study enrolled 14 Chinese patients who were diagnosed with NSCLC based on histopathological examination at Union Hospital, Tongji Medical College, Huazhong University of Science and Technology, between October 2018 and December 2021 and received anti-PD1 monoclonal antibody treatment. The inclusion criterion was a negative past history of cancer. An informed consent form was obtained from each participant. All patients underwent a follow-up. The clinical data were collected from the Union Hospital medical records database. The stage was classified according to the 8^th^ edition of TNM Staging of NSCLC by the International Association for the Study of Lung Cancer. The overall study protocol was approved by the Ethics Review Board of Wuhan Union Hospital, Huazhong University of Science and Technology, and the research was conducted in accordance with relevant ethical guidelines (2018-S271).

### 2.2 Study design

See **Figure 1**.

**Figure 1:**
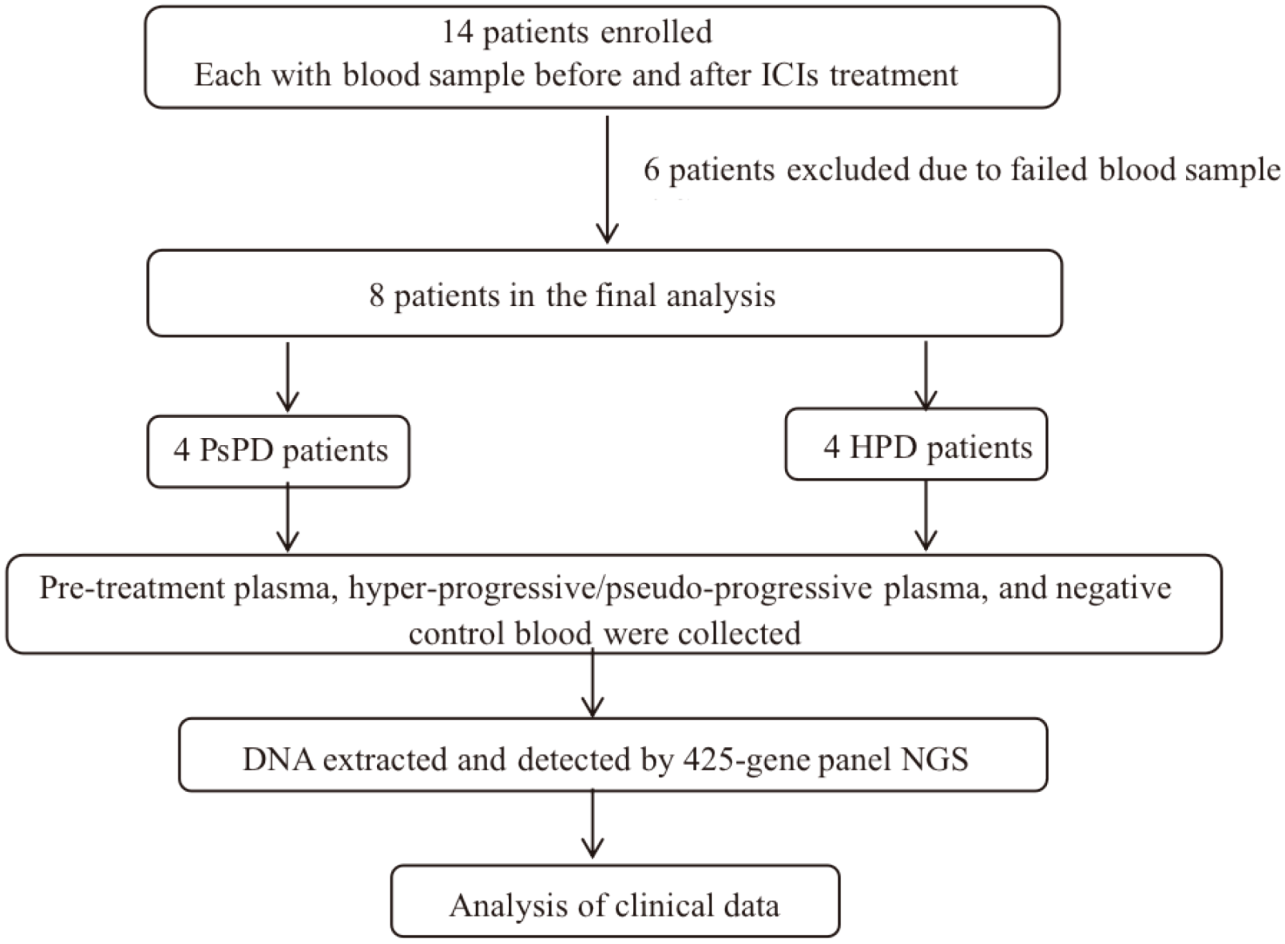
The design of this study.

### 2.3 Blood sample collection

A 10 mL blood sample was drawn and stored in a Cell-Free DNA Storage Tube (Streck 218962) at 25°C. Blood was centrifuged at 1,600 ×*g* for 10 min at 25°C to obtained plasma. Plasma was centrifuged at 1,600 ×*g* for 10 min at 4°C, and the supernatant was placed in a new tube. Plasma was separated from blood (no apparent hemolysis) within 72 h after blood collection and stored at −80°C until DNA isolation.

### 2.4 Sample preparation, DNA isolation, and sequencing

Circulating free DNA was extracted using the QIAamp Circulating Nucleic Acid Kit (QIAGEN, Hilden, Germany). Genomic DNA obtained from buccal swabs was prepared using the DNeasy Blood & Tissue kit (QIAGEN) as a control for germline mutations. DNA was quantified using the dsDNA HS Assay Kit (Life Technologies, Eugene, Oregon), according to the manufacturer’s recommendations. Sequencing libraries were prepared using the KAPA Hyper Prep Kit (KAPA Biosystems, Cape Town, South Africa), according to the manufacturer’s instructions for different sample types. Customized xGen lockdown probes (Integrated DNA Technologies, Coralville, IA, USA) targeting 425 tumor-related genes were used for hybridization enrichment (425 genes, **Supplementary Table 1**).

NGS was performed, followed by CLIA-certified and CAP-accredited assay validation at a centralized clinical testing center (Nanjing Geneseq Technology, Inc., Nanjing, China). The libraries were sequenced on a HiSeq 4000 NGS platform (Illumina, San Diego, CA, USA), and the sequencing data were analyzed to detect genomic alterations. The mean coverage depth was ~100X for controls and ~3,000X for circulating free DNA samples. The resultant sequences were analyzed for base substitutions, small insertions and deletions, copy number alterations (focal amplifications and homozygous deletions), and gene fusions/rearrangements.

### 2.5 Analysis of DNA sequences

Sequencing data were processed as described previously (11, 12). Briefly, the data were first subjected to demultiplexing and FASTQ file quality control to remove low-quality data or N bases. Qualified reads were mapped to the reference human genome GRCh37/hg19 using the Burrows-Wheeler Aligner (13) and default parameters to create Sequence Alignment/Map (SAM) files (14). Picard was used to convert the SAM files to compressed Binary Alignment Map (BAM) files, which were then sorted according to the chromosomal coordinates. The Genome Analysis Toolkit (15) was used to locally realign the BAM files at intervals with insertion/deletion (indel) mismatches and recalibrate the base quality scores of the reads in the BAM files. VarScan2 (16) was employed to detect single-nucleotide variations (SNVs) and indel mutations. The resulting mutation lists were further filtered through an internally collected list (1,000 normal samples) of recurrent artefacts on the same sequencing platform. SNVs and indels were further filtered based on the following parameters: (1) minimum read depth=20, (2) minimum base quality=15, (3) minimum variant supporting reads=5, (4) variant supporting reads mapped to both strands, (5) strand bias no greater than 10%, (6) if present in >1% population frequency in the 1000G or ExAC database, and (7) through an internally collected list of recurrent sequencing errors using a normal pool of 100 samples. Copy number variations (CNVs) were analyzed with the CNVkit (17) Depth ratios above 2 and below 0.6 were considered as gains and losses in CNVs, respectively. Variants that are predicted to shift the translational reading frame should be described using either a short or a long form p.(Arg97fs) and p.(Arg97Profs*23), respectively. For 'fsTer#'/'fs*#', it is specified that '#' indicates at which codon number the new reading frame ends with a stop codon. The number of the stop in the new reading frame is calculated starting at the first amino acid that is changed by the frame shift, ending at the stop codon (*#) (18).

### 2.6 TMB calculation

TMB was defined as the total number of missense mutations. In addition, we profiled TMB of these samples by a targeted NGS panel (Geneseeq) to evaluate its correlation with whole-exome sequencing (WES) results. Panel TMB was counted by summing all base substitutions and indels in the coding region of targeted genes, including synonymous alterations to reduce sampling noise and excluding known driver mutations as they are over-represented in the panel, as previously described (19, 20).

### 2.7 Response assessment

Treatment efficacy was assessed by the treating physician and another independent physician and classified according to the Response Evaluation Criteria in Solid Tumors (RECIST) version 1.1. Radiological evaluation of treatment efficacy by computed tomography (CT) was performed before treatment and on a schedule determined by each treating physician during treatment. HPD was defined as time-to-treatment failure <2 months, >50% increase in the tumor burden compared with that of pre-immunotherapy imaging, and >2-fold increase in the speed of progression. PsPD was defined as a partial response (PR) following RECIST-defined progressive disease (PD) during ICI treatment. The definition of PR in PsPD was assessed according to the changes observed from the time of PD and not from treatment initiation.

### 2.8 Statistical analysis

Figures were drawn using R-3.5.3 for Windows (32/64 bit), GraphPad Prism 8, and EXCEL. The difference for AF% of mutation gene between PsPD and HPD were analyzed by unpaired t test.

## 3 Results

### 3.1 Patient characteristics

Fourteen patients underwent eligibility assessment as described above, and eight patients were enrolled in this trial. The participants’ baseline characteristics are listed in **Supplementary Table 2**. All patients received anti-PD1 therapy, and their median age was 56 years (range, 27–72 years). Five of 14 (62.5%) received pembrolizumab (Keytruda^®^) treatment, while the others received sintilimab (Tyvyt^®^). The majority of patients were men (6/8, 75.0%) and had stage IV disease (7/8, 87.5%). Adenocarcinoma (6/8, 75.0%) was the predominant histopathological type, followed by squamous (1/14, 12.5%) or adenosquamous carcinoma (1/14, 12.5%). Four out of eight patients (50.0%) had a history of smoking, and 2/8 (25.0%) patients were non-smokers. Blood samples were collected from all patients at baseline and after image progression. NGS screening of plasma was performed for all patients (N=14), but only 8/14 were enrolled in the final analysis. Six patients were excluded owing to the poor quality of the blood sample. The median follow-up time was 4.0 months (range, 0.8–14.9 months), and the cut-off date was February 23, 2021. Till the cut-off date, 6/8 (75%) patients experienced disease progression, 4/8 (50%) had PsPD, and the others had HPD (**Supplementary Table 3**).

### 3.2 Mutational landscape

**Figure 2:**
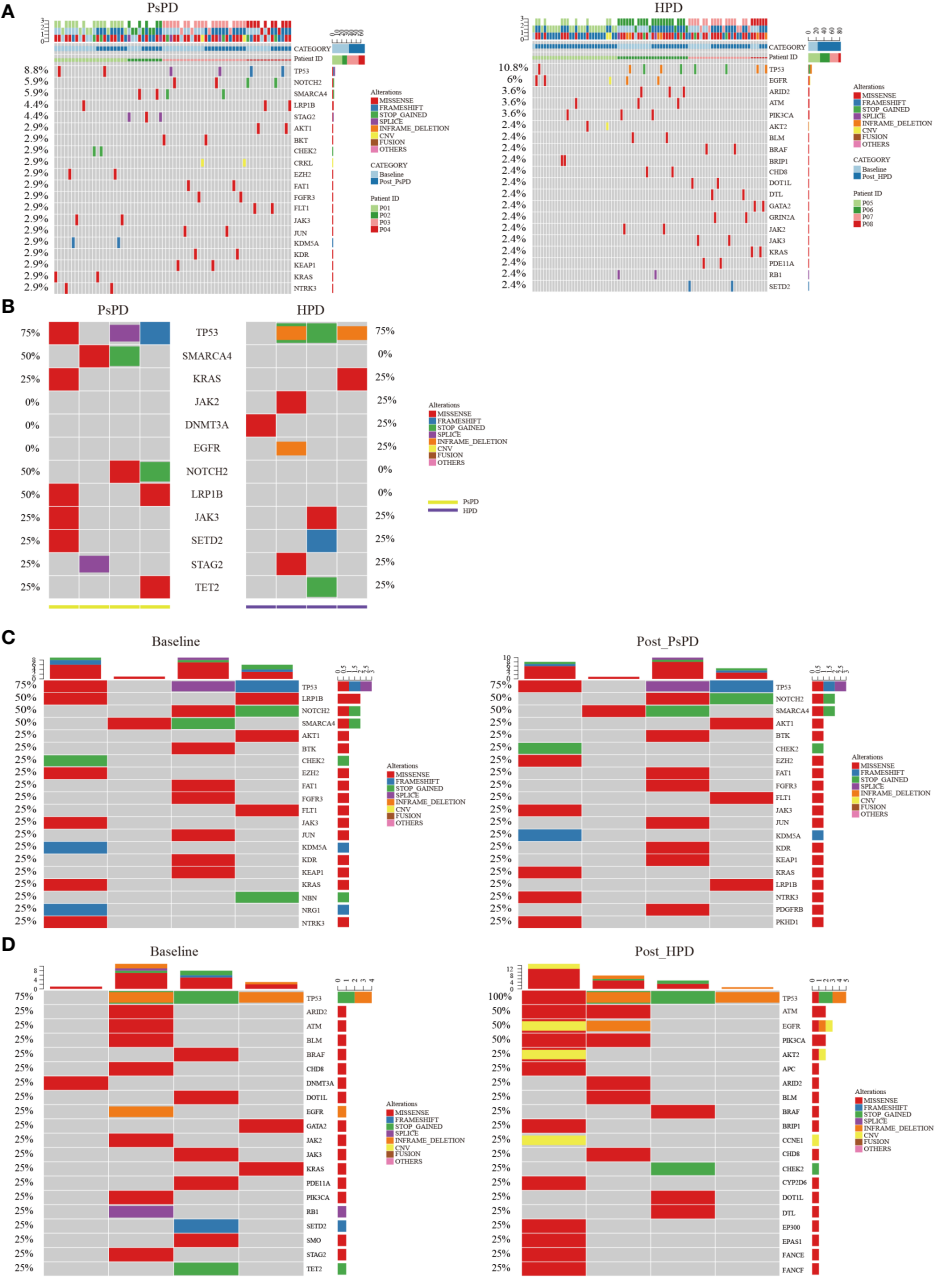
**(A)** Heat map of the top 20 mutant genes screened by plasma NGS in patients with PsPD/HPD (pooled data of baseline and after treatment). Left: PsPD, right: HPD. **(B)** Heat map of the 12 representative mutant genes at baseline in patients with PsPD/HPD. **(C)** Heat map of the top 20 mutant genes in patients with PsPD. Left: baseline, right: after PsPD. **(D)** Heat map of the top 20 mutant genes in patients with HPD. Left: baseline, right: after HPD. PsPD: pseudoprogression, HPD: hyperprogressive disease, NGS: next-generation sequencing.

All enrolled patients underwent a 425-panel NGS at baseline and after image progression. We identified a list of frequently mutated genes in NSCLC on the basis of these data; the 20 most common genes are illustrated in **Supplementary Figure 1**. Upon segregation of the PsPD and HPD groups, the mutational landscape of patients with the two responses showed a differential pattern. As shown in **Figure 2A**, the most frequently mutated genes in the PsPD group were TP53 (8.8%), NOTCH2 (5.9%), SMARCA4 (5.9%), LRP1B (4.4%), and STAG2 (4.4%), while TP53 (10.8%), EGFR (6.0%), ARD2 (3.6%), ATM (3.6%), and PIK3CA (3.6%) were the top 5 genes in the HPD group (**Supplementary Table 4**).

We compared the differences in the most common mutant genes between PsPD and HPD at baseline and the results were consistent with those of previous findings (**Figure 2B**). TP53, SMARCA4, and KRAS were positively related to immunotherapy efficacy, while JAK2 was negatively related to immunotherapy efficacy. We also observed EGFR and DNMT3A mutations in patients with HPD but not in those with PsPD, akin to the results of a previous study. Subsequently, we explored the genomic alterations after PsPD/HPD (**Figure 2C, D**). The top mutant gene frequency did not change considerably in patients with PsPD (**Table 1**), whereas the top mutant gene frequency increased significantly in patients with HPD (**Table 2**). As TP53 increased from 75.0% to 100.0%, ATM and EGFR increased from 25% to 50%.

**Table 1:** Gene mutation frequency change in PsPD.

Gene Rank	Before treatment		After PsPD	
	Gene name	Frequency	Gene name	Frequency
1	TP53	75%	TP53	75%
2	LRP1B	50%	NOTCH2	50%
3	NOTCH2	50%	SMARCA4	50%
4	SMARCA4	50%	AKT1	25%
5	AKT1	25%	BTK	25%
6	BTK	25%	CHEK2	25%
7	CHEK2	25%	EZH2	25%
8	EZH2	25%	FAT1	25%
9	FAT1	25%	FGFR3	25%
10	FGFR3	25%	FLT1	25%
11	FLT1	25%	JAK3	25%
12	JAK3	25%	JUN	25%
13	JUN	25%	KDM5A	25%
14	KDM5A	25%	KDR	25%
15	KDR	25%	KEAP1	25%
16	KEAP1	25%	KRAS	25%
17	KRAS	25%	LRP1B	25%
18	NBN	25%	NTRK3	25%
19	NRG1	25%	PDGFRB	25%
20	NTRK3	25%	PKHD1	25%

**Table 2:** Gene mutation frequency change in HPD.

Gene Rank	Before treatment		After HPD	
	Gene name	Frequency	Gene name	Frequency
1	TP53	75%	TP53	100%
2	ARID2	25%	ATM	50%
3	ATM	25%	EGFR	50%
4	BLM	25%	PIK3CA	50%
5	BRAF	25%	AKT2	25%
6	CHD8	25%	APC	25%
7	DNMT3A	25%	ARID2	25%
8	DOT1L	25%	BLM	25%
9	EGFR	25%	BRAF	25%
10	GATA2	25%	BRIP1	25%
11	JAK2	25%	CCNE1	25%
12	JAK3	25%	CHD8	25%
13	KRAS	25%	CHEK2	25%
14	PDE11A	25%	CYP2D6	25%
15	PIK3CA	25%	COT1L	25%
16	RB1	25%	DTL	25%
17	SETD2	25%	EP300	25%
18	SMO	25%	EPAS1	25%
19	STAG2	25%	FANCE	25%
20	TET2	25%	FANCF	25%

Herein, we investigated the single gene mutation pattern in PsPD and HPD before treatment and after image progression, as well as the variation tendency. It provided insights suggesting that alterations may reveal more information than a single mutation alone. Thus, the whole genomic spectrum was further analyzed.

### 3.3 Spectrum analysis of gene mutation

**Figure 3:**
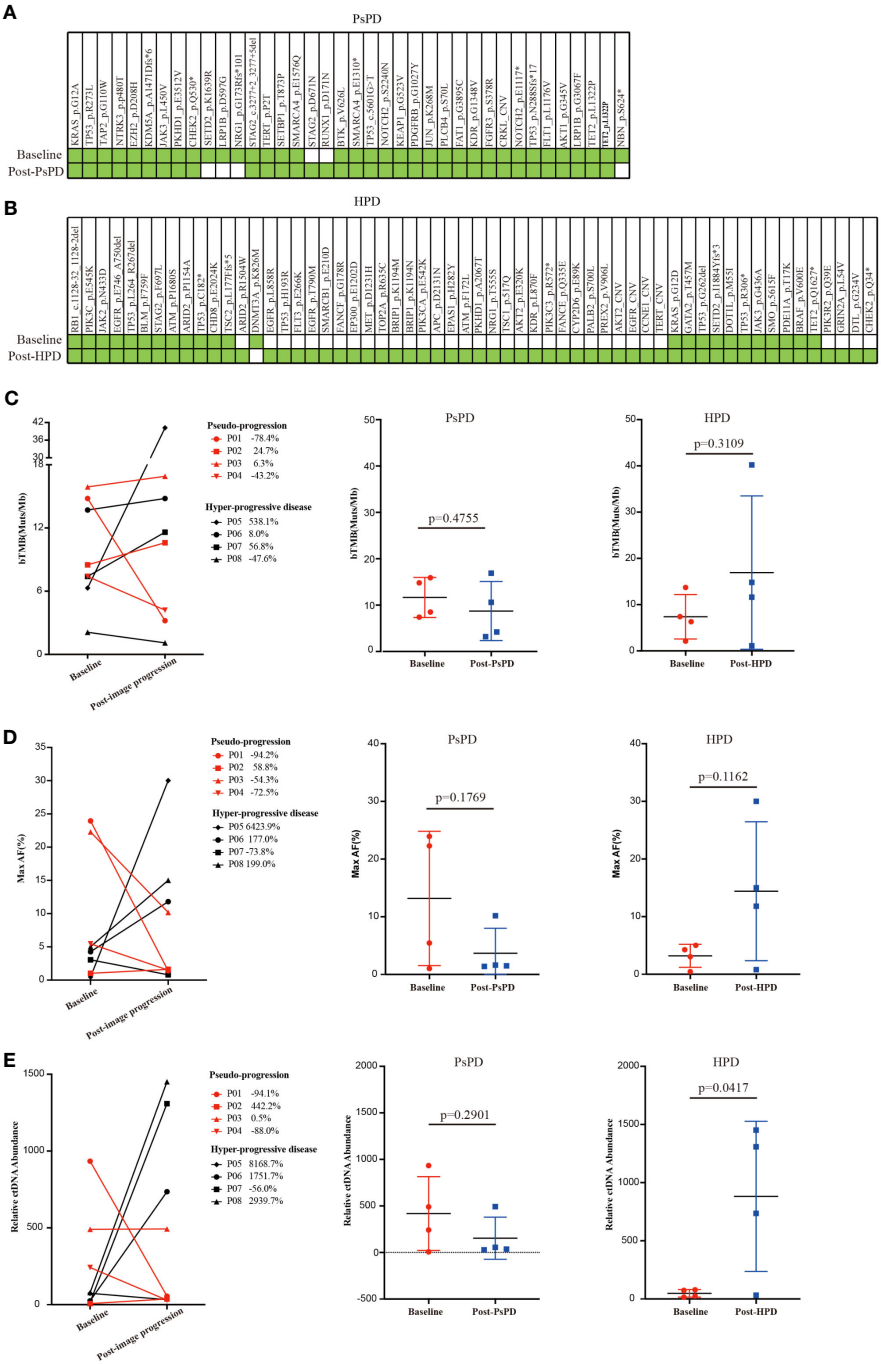
**(A)** Gene co-mutations in patients with PsPD at baseline and after imaging progression. **(B)** Gene co-mutations in patients with HPD at baseline and after imaging progression. **(C)** bTMB change from baseline to imaging progression in patients with PsPD/HPD left: connection diagram, middle: individual dotted diagram of bTMB at baseline, right: individual dotted diagram of bTMB after imaging progression. **(D)** Maximum AF% change from baseline to imaging progression in patients with PsPD/HPD left: connection diagram, middle: individual dots diagram of Max AF% at baseline, right: individual dotted diagram of maximum AF% after imaging progression. **(E)** Relative ctDNA abundance change from baseline to imaging progression in patients with PsPD/HPD. left: connection diagram, middle: individual dots diagram of relative ctDNA abundance at baseline, right: individual dots diagram of relative ctDNA abundance after imaging progression. Unpaired t test was used here. ct: circulating tumor, AF: allele frequency, bTMB: blood-based tumor mutational burden, PsPD: pseudoprogression, HPD: hyperprogressive disease, NGS: next-generation sequencing.

The aforementioned results provided a clue that the frequency of top mutational gene remained stable in patients with PsPD, while it increased significantly in patients with HPD. It also illustrated that the total number of mutated genes was elevated after HPD compared with that after PsPD. Based on the co-mutation gene data, we discovered that the frequency of co-mutated genes was higher in the PsPD group than in the HPD group (84.21% vs 38.33%) (**Supplementary Figure 2A-C**). The detailed gene spectrum is presented in **Figure 3A, B**, indicating that a higher co-mutation gene spectrum may indicate stable disease, while a lower co-mutation gene spectrum may suggest disease progression.

Several studies have found no association between blood-based TMB (bTMB) and the efficacy of immunotherapy. However, the association between change in the bTMB and the clinical outcomes of immunotherapy remains to be explored. Herein, we compared each patient’s bTMB at baseline and after image progression. The data revealed that bTMB was elevated in 75% and 50% of patients with HPD and PsPD, respectively (**Figure 3C**). In the HPD group, the greatest elevation reached 538.1%, whereas the highest value in the PsPD group was merely 24.7%. Interestingly, the maximum allele frequency (AF) increased significantly in 75% of patients with HPD, whereas only a mild elevation was observed in 25% of patients with PsPD (**Figure 3D**). The same pattern was also observed in circulating tumor DNA (ctDNA) abundance (**Figure 3E**). These results indicated that spectrum analysis of gene mutation data possessed greater efficacy in distinguishing between PsPD and HPD than single mutant gene analysis.

### 3.4 Alterations in gene mutation abundance

**Figure 4:**
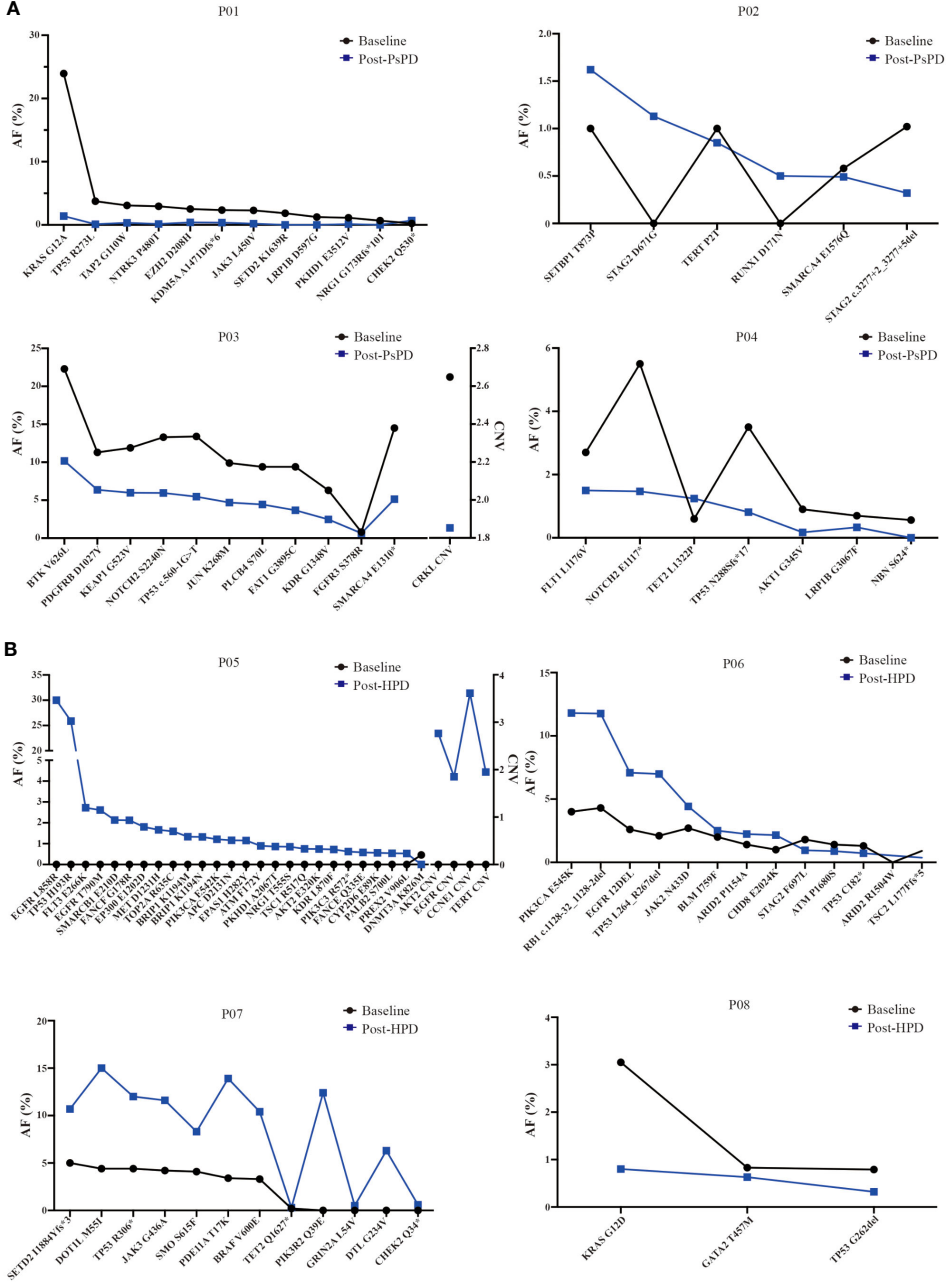
**(A)** AF% change in the total genomic mutation in patients with PsPD at baseline and after PsPD. The abbreviations represent different patients. **(B)** AF% change in the total genomic mutation in patients with HPD at baseline and after HPD. The abbreviations represent different patients. AF: allele frequency, CNV: copy number variation, PsPD: pseudoprogression, HPD: hyperprogressive disease.

Based on the NGS results, we discovered that the maximum AF for the top mutated genes decreased significantly in patients with PsPD (**Figure 4A**). On the contrary, it increased in patients with HPD (**Figure 4B**). The mutational abundance of the top three mutant genes, viz. TP53, KRAS, and SMARCA4, declined in patients with PsPD. These genes were also previously reported to be positive prognostic factors for immunotherapy. On the other hand, the mutational abundance of the top mutant genes such as EGFR, JAK2, and DNMT3A were elevated after progression in patients with HPD, which were previously reported to be negative prognostic factors of immunotherapy.

Thus, these data suggested that the genomic alteration after imaging progression can be a promising biomarker for distinguishing between PsPD and HPD in patients undergoing immunotherapy for NSCLC.

## 4 Discussion

ICIs confer tremendous survival benefits, especially in patients with NSCLC (1). However, they also pose great challenges for clinicians. In contrast to chemotherapy and radiotherapy, whose treatment response is evaluated using conventional radiographic methods, immunotherapy sometimes presents with unique treatment response patterns, such as PsPD and HPD (21). PsPD manifests with confusing imaging features, which may result in the loss of the potential benefits from ICIs. HPD, an extremely poor outcome of ICI therapy, often results in fatality, necessitating urgent attention. However, these atypical responses cannot be easily distinguished on conventional imaging, as the early stages of PsPD and HPD are both characterized by short-term enlargement of the tumor lesion (22). Some studies revealed that specific gene mutations may be associated with a corresponding unique response in patients (23); however, no single gene can accurately predict the efficacy of ICIs. NGS reveals more information about genomic mutation, whereas the evidence to link the whole genomic alteration and PsPD/HPD is lacking. Here, we have, for the first time, revealed the significant role of bTMB and shift in the mutational abundance before treatment and after imaging progression in the determination of PsPD/HPD in NSCLC. It highlights the clinical value of dynamic blood NGS monitoring to better understand the unique response patterns elicited by ICIs.

The incidence of HPD in NSCLC with ICIs treatment is reported to be 13.8%, highlighting a considerable variation in cases due to different causal factors (24). It is not triggered by a single factor, but by a series of events that occur simultaneously. T-regulatory cells lacking PD-1 signaling or tumor cells lacking PD-L1 have been shown to accelerate tumor development in HPD models (25, 26). Accumulating data demonstrate that high TGF-β is correlated with resistance to anti-PD-1/PD-L1 therapy, thus anti-TGF-β/PD-L1 bispecific antibodies such as YM101 and BiTP confer the resistance and exhibit enhanced antitumor activity in cancer treatment (27, 28). MDM2/MDM4 amplification may be associated with HPD (29, 30) as it promotes tumorigenesis directly or indirectly through the inhibition of p53. Pharmacological inhibitor results in an improvement in the antitumor immunity to anti-PD-1 treatment (31). EGFR is involved in immunotherapy-related resistance and HPD due to upregulation in the number of immunosuppressive receptors and induction of the secretion of cytokines (32–34). DNMT3A mutation is also related to poor outcomes with ICIs treatment in clinical research as well as EGFR and MDM2/4 mutations (35, 36). Loss-of-function mutations in JAK1/2 may play an important role in the lack of response to PD-1 inhibitors due to the reduced ability of immune T cells to recognize tumor cells. These mutations may result in the deficiency of T-cell infiltrates due to a deficit of chemokine production (37–39). PsPD is a rare phenomenon observed in <5% of cases of NSCLC (40). It is defined as the appearance of new lesions or tumor enlargement during therapy, followed by disease regression or stabilization at subsequent imaging (40). It indicates the true benefit of ICIs treatment, albeit with early pseudoprogression. Genetic studies investigating PsPD are even rarer, with most studies focusing on possible immunotherapy sensitivity. SMARCA4 mutation is reportedly associated with a favorable response to ICIs treatment in NSCLC. SMARCA4-mutant NSCLCs overlap genetically with frequent TP53 and KRAS mutations and a high TMB (41, 42). TP53 mutation has been proven to significantly increase the expression of immune checkpoints and activate the T-effector and interferon-γ signature, which contribute to the benefit conferred by ICIs. Specifically, the TP53/KRAS co-mutation subgroup manifested an exclusive increase in the expression of PD-L1 and mutational burden in the lung adenocarcinoma database, which also implies ICIs preference (43, 44). On the contrary, co-mutation of KRAS with STK11/LKB1 or KEAP1 indicated worse outcomes when ICIs are used for NSCLC therapy (44). Our study also found that EGFR, DNMT3A, and JAK2 were the most commonly mutated genes in the HPD group, while TP53, SMARCA4, and KRAS were the most commonly mutated genes in the PsPD group, consistent with previous research. Our study is rendered distinct by the fact that we used blood NGS to derive these results, which implies that blood gene testing could partly replace tissue detection.

However, some peculiarities were also observed during the course of this study. ATM belongs to the DNA damage repair pathway (DDR). Deficiency in the DDR activates innate immunity as well as tumor recognition of the adaptive immune system, leading to sensitivity to ICIs (45). However, our data showed that ATM mutation is observed in HPD, contrary to the previous hypothesis. Similarly, we discovered PIK3CA mutation in HPD, but other studies have shown that it leads to an elevation in the level of public neoantigens, indicating sensitivity to ICI treatment (46). These differences may be attributed to the different genetic backgrounds of various neoplasms and also the limited sample size of the current study. Moreover, previous evidence about the role of ATM and PIK3CA in immunotherapy has been derived from basic research, which stands in stark contrast to clinical practice. More studies are needed on this topic in the future.

As regular lung cancer gene testing recommended by the guidelines does not fully cover the above-mentioned genes, NGS can comprehensively detect gene mutations in patients who may receive immunotherapy to predict the effect of ICIs. However, the prediction and identification of specific response patterns such as PsPD and HPD by a single gene is still inaccurate. A vast amount of data suggest that TMB could be another predictor of ICI efficacy (47). Studies have discovered that the tissue TMB (tTMB) was not correlated with PD-L1 expression, but both are associated with the clinical benefits from ICIs (48). The TMB can be evaluated using various techniques with different thresholds and can be determined using tissues and blood (49). Evidence on its predictive value is conflicting. In the CheckMate 227 study, tTMB was proven to be a prospective biomarker for PFS. However, other randomized controlled trials have failed to show a survival benefit upon stratifying patients by tTMB, and their findings do not currently support the prognostic or predictive value of tTMB in NSCLC (9, 50). On the contrary, bTMB is valuable in predicting the ICI response (10, 51, 52). To date, the prognostic and predictive value of tTMB or bTMB remains elusive, and evidence of their direct association with PsPD and HPD is lacking.

Our data showed that the bTMB before treatment partly represents the specific ICIs response, consistent with a previous study. Surprisingly, the absolute bTMB after imaging progression shows a vast difference between PsPD and HPD. More interestingly, comparison of the pre- and post-treatment values revealed that bTMB elevation was significantly higher in the HPD group than that in the PsPD group. Not only does it yield the bTMB, but also provides information about AF and ctDNA abundance. The fold change differences between PsPD and HPD were even more significant with respect to the changes in AF% and relative ctDNA abundance. In the HPD group, P08 progressed rapidly after treatment, and the interval between the two tests was only 3 weeks; therefore, the data may not represent the actual situation. If this patients’ data are excluded, it can be seen that the ratios of bTMB, maximum AF%, and relative ctDNA abundance increased significantly in all patients with HPD after progression. Patients with PsPD showed the opposite result. These results may indicate the association of the decrease in gene mutation abundance with predicting the decrease in the tumor burden; additionally, these findings underscore the potential value and superiority of dynamic blood genomic alteration in predicting the outcomes of ICIs treatment.

This study provides a novel method to distinguish HPD and PsPD in NSCLC patients receiving anti-PD1 treatment. To the best of our knowledge, this is the first study to illustrate a dynamic comparison of the whole genomic alteration by blood NGS to differentiate PsPD and HPD, thereby providing clinical evidence to evaluate the outcome of immunotherapy. The scarcity of eligible patients meeting our criteria posed a challenge in this study. However, given the clinical significance of our findings, we are committed to expanding our sample size for validation. We are currently employing ambulatory blood NGS detection on patients undergoing treatment at our center, and the preliminary results align with those presented here. We are also exploring cost-effective prediction methods, such as dynamic monitoring of ctDNA and CTC as other previous studies (53). Existing research has indeed highlighted disparities in gene mutation frequencies among different racial groups, particularly in targeted therapy for lung cancer (54). Immunotherapy has also shown the different efficacy across ethnicities (55). We acknowledge that racial differences in genetic testing evaluation warrant attention. It is important to note that our study focused on overall gene-level changes rather than specific gene mutations, which may not be clinically significant in this context. Nonetheless, we eagerly anticipate the accumulation of data from diverse regions and research centers to validate our findings, with the aim of benefits to lung cancer patients globally, transcending geographical boundaries, and not limited to the Chinese population. Genomic change may be associated with the adverse effects of ICIs, which is also a difficult problem. Unfortunately, the adverse effects were not included here. It is a practical issue that needs to be addressed in future research.

In conclusion, for patients with NSCLC receiving anti-PD1 treatment, the PsPD group showed a significant reduction in the bTMB, AF%, and relative ctDNA abundance in the whole genome as well as a decrease in the expression of all mutational genes, while the opposite was observed in the HPD group. For the patients with image progression shortly after receiving anti-PD1 treatment despite better symptoms, a second blood NGS was preferred. These results broaden the scope of the dynamic genome-wide spectrum in differentiating PsPD from HPD and provide preliminary data to support the continuous blood-based NGS detection during ICI therapy for NSCLC.

## Data availability statement

The original contributions presented in the study are included in the article/**Supplementary Material**. Further inquiries can be directed to the corresponding authors.

## Ethics statement

The studies involving humans were approved by the Ethics Review Board of Wuhan Union Hospital, Huazhong University of Science and Technology and the research was conducted in accordance with relevant ethical guidelines (2018-S271). The studies were conducted in accordance with the local legislation and institutional requirements. The participants provided their written informed consent to participate in this study.

## Author contributions

XD: Conceptualization, Investigation, Writing – Review and Editing, Supervision, Funding acquisition. SZ: Methodology. YB: Software, Resources. FT: Validation, Data curation. RZ: Formal analysis, Writing – Original Draft, Writing – Review and Editing. NY: Investigation, Supervision. YZ: Visualization. RGZ: Project administration. All authors contributed to the article and approved the submitted version.
