# Supplementary figures and images for: Genomic alterations associated with pseudoprogression and hyperprogressive disease during anti-PD1 treatment for advanced non-small-cell lung cancer
Source: Front Oncol. 2023 Nov 9;13:1231094. doi: 10.3389/fonc.2023.1231094 (PMC10667039; doi:10.3389/fonc.2023.1231094)

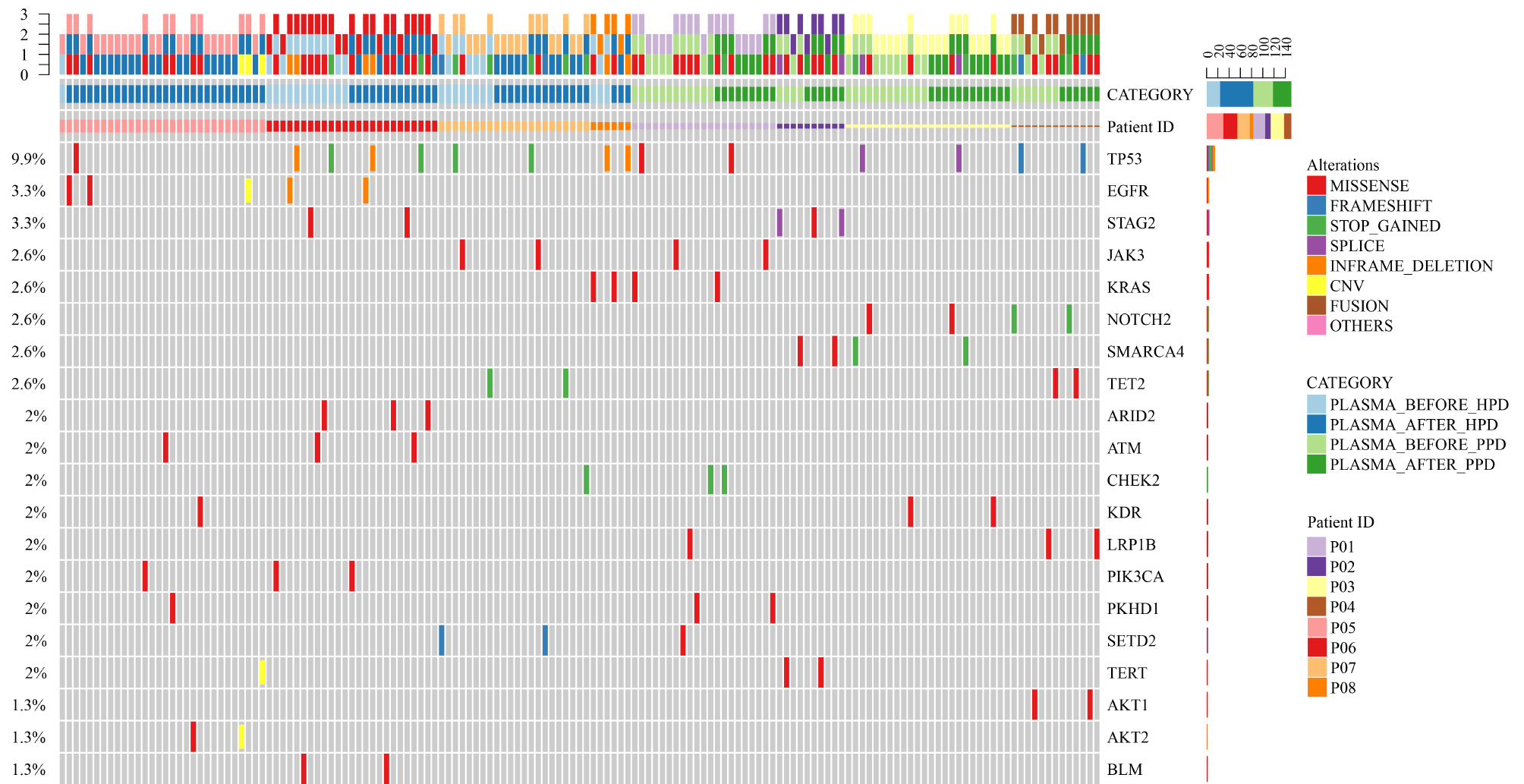

Supplement: Supplementary file 1 [file Image_1.tif]

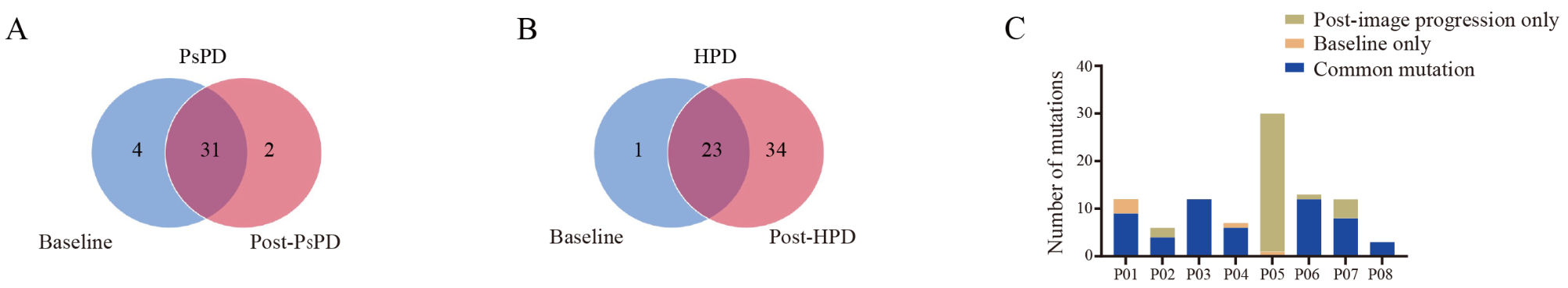

Supplement: Supplementary file 2 [file Image_2.tif]

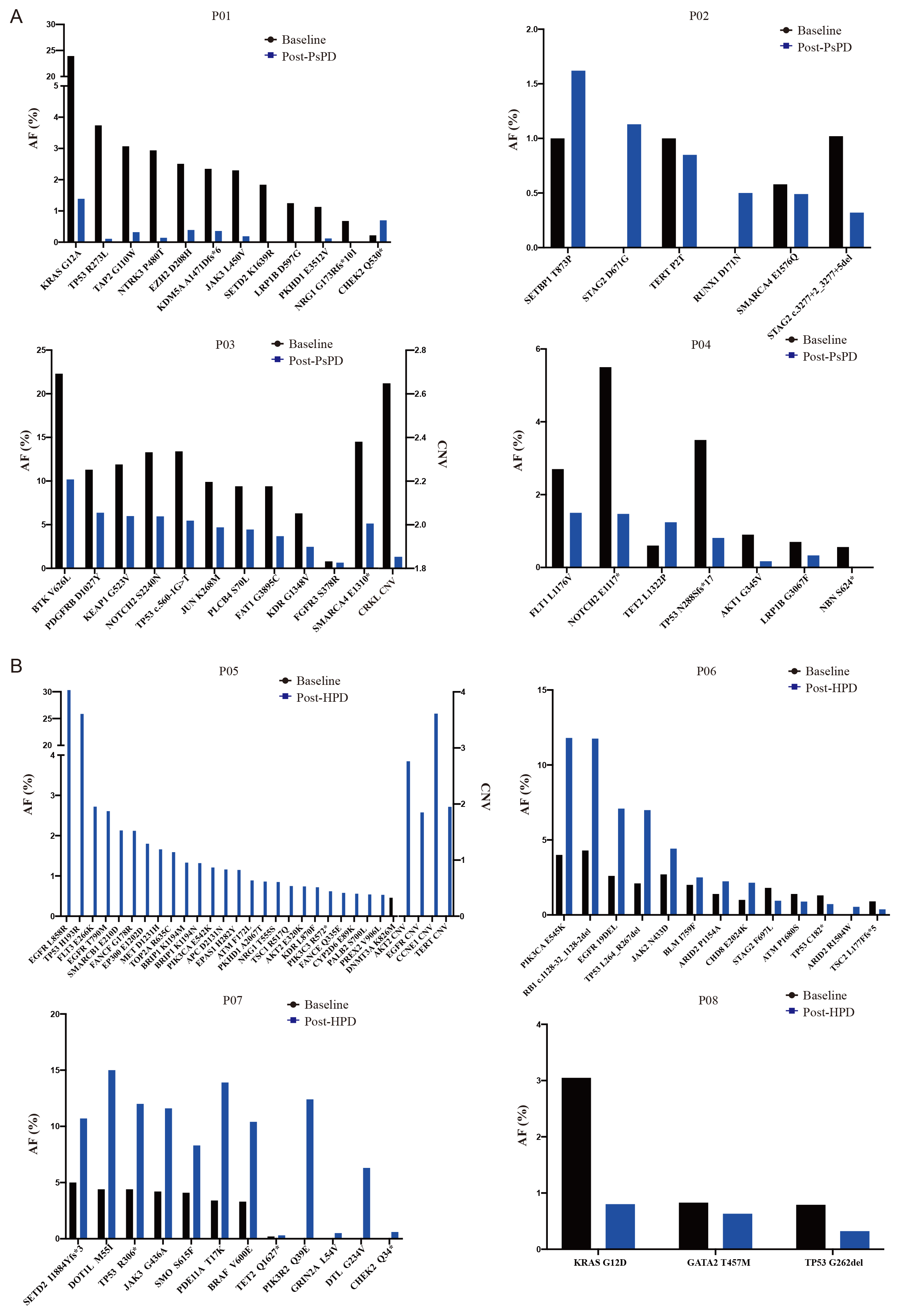

Supplement: Supplementary file 3 [file Image_3.tif]
