# Supplementary material for: Genomic alterations associated with pseudoprogression and hyperprogressive disease during anti-PD1 treatment for advanced non-small-cell lung cancer
Source: Front Oncol. 2023 Nov 9;13:1231094. doi: 10.3389/fonc.2023.1231094 (PMC10667039; doi:10.3389/fonc.2023.1231094)
Supplement: Supplementary file 4 [file Table_1.docx]

**Supplemental Table 1. Custom panel genes analyzed**

| 425 genes |
| --- |
| *ABCB1、AIP、AKT1、AKT2、AKT3、ALK、AMER1、APC、AR、ARAF 、ARID1A、ARID2 、ASCL4、ATM、ATR 、ATRX、AURKA、AURKB、AXIN2、AXL、B2M、BAD、BAK1、BAP1、BARD1 、BAX、BCL2、BLM、BMPR1A、BRAF、BRCA1、BRCA2、BRD4 、BRIP1、BTG2、BTK、BUB1B、c11orf30、CBL、CBLB、CCND1、CCNE1、CD274 、CDA、CDC73、CDH1、CDK12、CDK4、CDK6、CDK8、CDKN1A、CDKN1B、CDKN1C、CDKN2A、CDKN2B、CDKN2C、CEBPA、CEP57、CHD4 、CHEK1、CHEK2、CREBBP、CRKL 、CSF1R、CTCF 、CTLA4、CTNNB1、CYLD、CYP2A13、CYP2A6、CYP2A7、CYP2D6、DAXX、DDR2、DENND1A、DICER1、DLL3、DNMT3A、DPYD、DUSP2、EGFR、EP300、EPAS1、EPCAM、EPHA2、EPHA3 、ERBB2 、ERBB3、ERBB4、ERCC1、ERCC2、ERCC3、ERCC4、ERCC5、ESR1 、EXT1、EXT2、EZH2、FANCA、FANCC、FANCD2、FANCE、FANCF、FANCG、FANCI、FANCL、FAT1、FBXW7、FGF19 、FGFR1、FGFR2、FGFR3、FGFR4、FH、FLCN、FLT1 、FLT3、FLT4、GATA1、GATA2、GATA3、GATA4 、GATA6 、GNA11 、GNAQ 、GNAS 、GRIN2A、GRM3 、GSTM1、GSTT1、HDAC2、HGF、HNF1A、HRAS、IDH1、IDH2、IFNG、IFNGR1、IGF1R、IGF2 、IKBKE、IL7R、INPP4B、IRF2 、JAK1、JAK2、JAK3、JUN、KDM5A、KDR 、KEAP1、KIT、KITLG、KLLN、KMT2A 、KMT2B、KRAS、LHCGR、LMO1、LYN、LZTR1 、MAP2K1 、MAP2K2 、MAP2K4、MAP3K1、MAX、MCL1、MDM2、MDM4、MED12、MEF2B、MEN1、MET、MGMT、MITF、MLH1、MLH3、MPL、MRE11A 、MSH2、MSH6、MTOR、MUTYH、MYC、MYCL、MYCN、MYD88、NAT1、NBN、NF1、NF2、NFKBIA 、NKX2-1、NOTCH1、NOTCH2、NPM1、NRAS、NRG1、NSD1、NTRK1 、PAK3、PALB2、PALLD、PARK2 、PARP1、PARP2、PAX5、PBRM1、PDCD1 、PDCD1LG2 、PDE11A、PDGFRA、PDGFRB、PDK1 、PGR、PHOX2B、PIK3C3、PIK3CA、PIK3R1、PIK3R2、PKHD1、PLK1、PMS1、PMS2、POLD1 、POLE、POLH、POT1、PPARD、PPP2R1A、PRDM1、PRF1、PRKACA、PRKAR1A、PRKCI 、PRSS1、PTCH1、PTEN、PTK2、PTPN11、PTPN13、QKI 、RAC1 、RAC3、RAD50、RAD51、RAD51B、RAD51C、RAD51D、RAD54L、RAF1、RARA、RB1、RECQL4、RET、RHOA、RICTOR、RNF43 、ROS1、RPTOR、RRM1、RUNX1、SBDS、SDHA 、SDHB、SDHC、SDHD、SETD2 、SGK1、SMAD2、SMAD3 、SMAD4、SMARCA4、SMARCB1、SMO、SOX2、SPOP 、SPRY4、SRC、STAG2、STAT3 、STK11、STMN1、SUFU、TAP1、TAP2、TEK、TERC、TET2、TGFBR2、THADA、TMEM127、TNFAIP3、TNFRSF11A、TNFRSF14、TNFSF11、TOP1、TOP2A、TP53、TPMT、TSC1、TSC2、TSHR、TTF1、TUBB3、UGT1A1、VEGFA、VHL、WAS、WISP3 、WRN、WT1、XPA、XPC、YAP1、ZNF217 、ZNF703、ABCB4、ABCC2 、ADH1A、ADH1B、ADH1C、ALDH2、ARID1B、ARID5B、ASXL1、ATF1、ATIC、BAI3、BCL2L11 、BCR、BIRC3、CASP8、CD74、CDK10、CUL3、CUX1、CXCR4、CYP19A1、CYP2B6*6、CYP2C19*2、CYP2C9*3、CYP3A4*4、CYP3A5、DHFR、EML4、EPHA5、EPHB2、ERBB2IP、ETV1、ETV4、ETV6、EWSR1、FANCM、FOXA1、FOXP1、FRG1、GRM8、GSTM4、GSTM5、GSTP1、HDAC9、HLA-A、HNF1B 、HSD3B1、IKZF1、JARID2、KDM6A、KIF1B、KIF5B 、KMT2C、KMT2D 、LRP1B、MAP3K4、MAP4K3、MECOM、MLLT1、MLLT3、MLLT4、MTHFR、MYH9、NCOR1、NFE2L2、NKX2-4、NOTCH3、NQO1、NTRK2、NTRK3、PLAG1、POLD3 、PRKACG、PRKDC、PRSS3、PTPRD、RARG、RASGEF1A、RELN、RUNX1T1、SDC4、44083、SETBP1、SF3B1 、SLC34A2、SLC3A2、SLC7A8、SMAD7 、SOS1、SOX1、SOX14、SOX21、SRY、STT3A、TEKT4、TERT 、TMPRSS2 、TNFRSF19、TP63、TUBB4A、TUBB4B、TUBB6、TYMS、U2AF1、VAMP2、XRCC1、ZNF2* |
