# Supplementary material for: Genomic alterations associated with pseudoprogression and hyperprogressive disease during anti-PD1 treatment for advanced non-small-cell lung cancer
Source: Front Oncol. 2023 Nov 9;13:1231094. doi: 10.3389/fonc.2023.1231094 (PMC10667039; doi:10.3389/fonc.2023.1231094)
Supplement: Supplementary file 5 [file Table_2.docx]

**Supplemental Table 2 Baseline characteristics of included Patient**

|  | | **Patients（n=8）** |
| --- | --- | --- |
| **Characteristics** | | **No. Patients (%)** |
| Age(years) | ＜65 | 6(75.0%) |
|  | ≥65 | 2(25.0%) |
|  | Median（range） | 56(27-72) |
| Sex | Male | 6(75.0%) |
|  | Female | 2(25.0%) |
| Clinical Stage | III | 1(12.5%) |
|  | IV | 7(87.5%) |
| Histology-Based Subtyping | Adenocarcinoma | 6(75.0%) |
|  | Squamous | 1(12.5%) |
|  | Adenosquamous Carcinoma | 1(12.5%) |
| Smoking Status | Never-Smoker | 2(25.0%) |
|  | Former-Smoker | 4(50.0%) |
|  | Unknow | 2(25.0%) |
| Immunotherapy drugs | Pembrolizumab | 5(62.5%) |
|  | Sintilimab | 3(37.5%) |
| Immunotherapy status | pseudo-progression | 4(50%) |
|  | hyper-progressive disease | 4(50%) |
