# Supplementary material for: Genomic alterations associated with pseudoprogression and hyperprogressive disease during anti-PD1 treatment for advanced non-small-cell lung cancer
Source: Front Oncol. 2023 Nov 9;13:1231094. doi: 10.3389/fonc.2023.1231094 (PMC10667039; doi:10.3389/fonc.2023.1231094)
Supplement: Supplementary file 6 [file Table_3.docx]

**Supplemental Table 3 Patients’ treatment detail (N = 8)**

| **Patients' code** | **Response pattern** | **Drug** | **Prgression** | **PFS (months)** |
| --- | --- | --- | --- | --- |
| LIYO | pseudo-progression | Sintilimab | Yes | 12.67 |
| LGQI | pseudo-progression | Pembrolizumab | No | 14.93 |
| YZFE | pseudo-progression | Pembrolizumab | Yes | 5.93 |
| ZQMI | pseudo-progression | Sintilimab | No | 4.13 |
| XYGU | hyper-progressive disease | Pembrolizumab | Yes | 0.83 |
| MQRO | hyper-progressive disease | Sintilimab | Yes | 1.4 |
| ZHSHR | hyper-progressive disease | Pembrolizumab | Yes | 2 |
| ZHFSH | hyper-progressive disease | Pembrolizumab | Yes | 1.5 |
