# Supplementary material for: Genomic alterations associated with pseudoprogression and hyperprogressive disease during anti-PD1 treatment for advanced non-small-cell lung cancer
Source: Front Oncol. 2023 Nov 9;13:1231094. doi: 10.3389/fonc.2023.1231094 (PMC10667039; doi:10.3389/fonc.2023.1231094)
Supplement: Supplementary file 7 [file Table_4.docx]

**Supplemental Table 4 Top 20 mutant gene in PsPD and HPD before treatment**

| **Rank** | PPD | | HPD | |
| --- | --- | --- | --- | --- |
|  | Gene Name | Frequency | Gene Name | Frequency |
| **1** | TP53 | 8.5% | TP53 | 10.8% |
| **2** | NOTCH2 | 5.9% | EGFR | 6.0% |
| **3** | SMARCA4 | 5.9% | ARID2 | 3.6% |
| **4** | LRP1B | 4.4% | ATM | 3.6% |
| **5** | STAG2 | 4.4% | PIK3CA | 3.6% |
| **6** | AKT1 | 2.9% | AKT2 | 2.4% |
| **7** | BTK | 2.9% | BLM | 2.4% |
| **8** | CHEK2 | 2.9% | BRAF | 2.4% |
| **9** | CRKL | 2.9% | BRIP1 | 2.4% |
| **10** | EZH2 | 2.9% | CHD8 | 2.4% |
| **11** | FAT1 | 2.9% | DOT1L | 2.4% |
| **12** | FGFR3 | 2.9% | DTL | 2.4% |
| **13** | FLT1 | 2.9% | GATA2 | 2.4% |
| **14** | JAK3 | 2.9% | GRIN2A | 2.4% |
| **15** | JUN | 2.9% | JAK2 | 2.4% |
| **16** | KDM5A | 2.9% | JAK3 | 2.4% |
| **17** | KRD | 2.9% | KRAS | 2.4% |
| **18** | KEAP1 | 2.9% | PDE11A | 2.4% |
| **19** | KRAS | 2.9% | RB1 | 2.4% |
| **20** | NTRK3 | 2.9% | SETD2 | 2.4% |
